# A Decision Support Framework for Automated Screening of Diabetic Retinopathy

**DOI:** 10.1155/IJBI/2006/45806

**Published:** 2006-02-02

**Authors:** P. Kahai, K. R. Namuduri, H. Thompson

**Affiliations:** ^1^ Department of Electrical & Computer Engineering, Wichita State University, Wichita, KS 67260-0044, USA; ^2^ LSU Eye Center, Louisiana State University, New Orleans, LA 70112, USA

## Abstract

The early signs of diabetic retinopathy (DR) are depicted by microaneurysms among other signs. A prompt diagnosis when the disease is at the early stage can help prevent irreversible damages to the diabetic eye. In this paper, we propose a decision support system (DSS) for automated screening of early signs of diabetic retinopathy. Classification schemes for deducing the presence or absence of DR are developed and tested. The detection rule is based on binary-hypothesis testing problem which simplifies the problem to yes/no decisions. An analysis of the performance of the Bayes optimality criteria applied to DR is also presented. The proposed DSS is evaluated on the real-world data. The results suggest that by biasing the classifier towards DR detection, it is possible to make the classifier achieve good sensitivity.


Hindawi Publishing Corporation
International Journal of Biomedical Imaging
Volume 2006, Article ID 45806, Pages 1–8
DOI 10.1155/IJBI/2006/45806

### A Decision Support Framework for Automated Screening of Diabetic Retinopathy

**P. Kahai,[1] K. R. Namuduri,[1] and H. Thompson[2]**

[1] *Department of Electrical & Computer Engineering, Wichita State University, Wichita, KS 67260-0044, USA*
[2] *LSU Eye Center, Louisiana State University, New Orleans, LA 70112, USA*

Received 8 August 2005; Accepted 8 October 2005

Recommended for Publication by Jie Tian

The early signs of diabetic retinopathy (DR) are depicted by microaneurysms among other signs. A prompt diagnosis when the disease is at the early stage can help prevent irreversible damages to the diabetic eye. In this paper, we propose a decision support system (DSS) for automated screening of early signs of diabetic retinopathy. Classification schemes for deducing the presence or absence of DR are developed and tested. The detection rule is based on binary-hypothesis testing problem which simplifies the problem to yes/no decisions. An analysis of the performance of the Bayes optimality criteria applied to DR is also presented. The proposed DSS is evaluated on the real-world data. The results suggest that by biasing the classifier towards DR detection, it is possible to make the classifier achieve good sensitivity.

Copyright © 2006 P. Kahai et al. This is an open access article distributed under the Creative Commons Attribution License, which permits unrestricted use, distribution, and reproduction in any medium, provided the original work is properly cited.

### 1. INTRODUCTION

According to the American Diabetes Association, 18.2 million of the American population, which constitutes 6.3% of the total population, have diabetes. In the United States alone, diabetes is responsible for 8% of legal blindness, making it the leading cause of blindness in people between 20 and 74 years of age [1–3]. Researchers including the authors of this paper [4] have therefore suggested an automated screening system for diabetic retinopathy for prompt diagnosis. Since the disorders exhibited in the early stage do not affect the vision, detection of the disease right at its onset can be done only if regular eye examination of the diabetic patients is performed.

This paper proposes an automated screening system that would detect early signs of nonproliferative diabetic retinopathy (NPDR). The contributions of this paper are twofold: (a) automated detection methods based on image processing for identifying lesions related to DR and (b) a decision support system (DSS) for automated DR screening. Classification schemes for deducing the presence or absence of DR are developed and tested. A univariate approach has been devised to test the suitability of the classification mechanism with respect to the detection of retinopathy. Classification is performed by test data subjected to unsupervised learning. This approach has been developed for one particular feature but the feature space can be extended depending on the number of disorders needed to be detected. The detection rule is developed based on binary-hypothesis testing problem which simplifies the problem to yes/no decisions. An analysis of the performance of the Bayes optimality criteria applied to DR is also presented.

The test data for the classification scheme is composed of real-world retinal images obtained from Lions Eye Research Center at LSU, New Orleans. The data contains retinal images that belong to either background retinopathy, maculopathy, or preproliferative retinopathy. The DSS framework focuses mainly on microaneurysms as these are the early signs of DR and are present at all the stages as the disease progresses from mild to severe NPDR. The DR screening results obtained from the DSS are compared with the physician's diagnosis to measure the system's sensitivity. The results suggest that by biasing the classifier towards DR detection, it is possible to achieve 100% sensitivity, although at reduced specificity of 67%. Since sensitivity implies the presence of abnormality, this biasing towards sensitivity is reasonable.

The organization of the paper is as follows. Section 2 describes the background and related work. Section 3 presents the decision framework adopted for the detection of microaneurysms followed by the experimental results and conclusions in Sections 4 and 5, respectively.

### 2. BACKGROUND AND RELATED WORK

Diabetic retinopathy is a progressive disease and the condition may advance from mild retinopathy to severe proliferative retinopathy. Diabetes Control and Complications Trial (DCCT) [5] and the UK Prospective Diabetes Study (UKPDS) [6] concluded that blood glucose and blood pressure control can slow down retinopathy.

### 2.1. DR related disorders and stages

Diabetic retinopathy can be broadly classified as nonproliferative diabetic retinopathy (NPDR) and proliferative retinopathy (PDR).The European Association for Study of Diabetic Complications (EASDEC) [7] has characterized the stages of diabetic retinopathy as follows.

(i) *Background retinopathy*. This condition is often present without any visual impairment and can therefore go unnoticed if dilated eye exam is not undertaken by a diabetic patient after regular intervals. Findings in the retina show microaneurysms and exudates that are enlarged tiny blood vessels (dots) and tiny haemorrhages or leaky areas (blots), respectively, on the surface of the retina.

(ii) *Maculopathy*. Areas of leakage develop in the retina and the retina becomes boggy. The leak can continue to enlarge. The waterlogging can affect the central part of retina, macula, and eventually affect the vision.

(iii) *Preproliferative retinopathy*. A large number of haemorrhages and microaneurysms are exhibited and IRMA along with venous beading is seen. The condition is called "preproliferative" as it usually progresses to proliferative retinopathy, when "new vessels" develop.

(iv) *Proliferative retinopathy*. Proliferative diabetic retinopathty (PDR) has the greatest risk of visual loss. The condition is characterized by the development of new, abnormal vessels (neovascularisation) near the optic nerve and haemorrhages in the vitreous humor and in front of the retina. The neovascular vessels are weak and can bleed into the vitreous humor of the eye leading to permanent complications.

The stages that precede the proliferative retinopathy constitute nonproliferative diabetic retinopathy (NPDR). A prompt diagnosis at the early stage of the diabetic retinopathy can help prevent severe damages to the retina of a diabetic patient.

### 2.2. Automated DR screening system

An automated screening system for diabetic retinopathy consists of the following three stages as described below. The first stage involves image-based feature detection and analysis, that is, identifying the patterns of interest using image processing methods. Image segmentation, edge/boundary detection, shape, and texture analysis are some of the techniques commonly used in image processing for pattern detection purposes. Feature analysis can be carried out on the original image or in the transform domain.

The next stage involves representing the features in the feature space and analyzing the features jointly in order to characterize the image, as a whole, in terms of retinal disorders. The abnormal features detected using the feature analysis provide very useful information such as the location, size, center, and other geometrical aspects of the features. This information needs to be analyzed in the feature space in order to reduce the ambiguities that are common in any image analysis.

The final stage involves developing a classification scheme that classifies the given retinal image based on the abnormalities present in the retinal images and the severity that they exhibit. Confidence levels need to be estimated using statistical methods for all the estimates. Discriminant functions and algorithms for distinguishing images based on the features present in the image need to be developed.

While significant research [8, 9] is being carried out in the field of extracting vessels and abnormalities from retinal images, a comprehensive framework for automated screening using statistical framework and feature analysis has not been developed so far in the research community.

### 2.3. Feature detection using image processing techniques

Researchers [10–14] have approached the problem of feature detection in varied ways. A modular system developed in [15] makes use of a large database of images where features have been identified by the physicians. This database is later used to detect similar features in new images. The recognition process employs unsupervised learning mechanism and the classification phase uses supervised learning.

Another technique proposed in [16] automatically detects and distinguishes between different lesions (hard exudates, cotton-wool spots, and haemorrhages) after image enhancement. The image is enhanced by taking the difference between the background illumination and an edge detection operator.

An automated system such as the one proposed in [17, 18] can be used to substitute a trained observer to identify and quantify microaneurysms. In this system, a combination of shade correction, matched filters, and shape algorithms are used to detect microaneurysms in fluorescein angiogram.

A comparative microaneurysm digital detection system is described in [10, 19, 20]. This system registers retinal images of the same eye subjected to a series of studies by focusing on the regions centered around the fovea and provides a comparative result for the count of microaneurysms.

Detection of microaneurysms in digital angiograms of the eye fundus is proposed in [21]. This system determines the count of microaneurysms by first enhancing the retinal image and then employing object segmentation.

### 3. PROPOSED DECISION SUPPORT SYSTEM

In this paper, three different classification schemes based on the Bayesian framework, namely, the likelihood ratio test, maximum a posteriori detector, and Bayes detector, are presented. The decision framework considers the following four possible outcomes in order to analyze the performance of the classification schemes.

(i) Correct accept/hit (sensitivity): a person affected with diabetic retinopathy has a true diagnosis.
(ii) False accept/false alarm: a normal person diagnosed with diabetic retinopathy.
(iii) False reject/miss: an affected person diagnosed as normal.
(iv) Correct reject (specificity): a normal person classified as unaffected.

The erroneous classifications are given by false accept (FA) and false reject (FR) which are referred to as type-I and type-II errors [22]. In relation to diabetic retinopathy application, the cost associated with each of these would be governed by the amount of harm caused by a misdiagnosis. The repercussions incurred in categorizing a person affected with diabetic retinopathy as normal are obviously more than the converse. Some statisticians refer to the cost function as the loss function. The cost or the loss associated with any misclassification is directly proportional to the severity induced by the error.

Considering a univariate case and analyzing each feature independently, the probability density functions obtained for the case wherein microaneurysms are present and the case where they are absent are shown are Figure 1. The data is composed of 25 normal images and 23 affected images of the retina selected from a large pool of images.

It can be seen that the region of overlap ranges from a feature value of 0.1 to 0.375. The boundary value occurs at feature value of 0.3. At the boundary value, decision can be taken for either case. But the loss function can be such that the loss associated with normal classification is more than that with abnormal classification. Thus, in order to minimize the risk involved in misclassification, all the cases at the boundary value can be classified as abnormal.

The region under the abnormal probability density function ranging from 0.1 to 0.3 corresponds to the false reject rate where an abnormal person is classified as unaffected. And the region under the normal probability density function ranging from 0.375 to 0.4 corresponds to the false accept rate where a normal person is categorized as affected. The area under the false accept region gives the false alarm probability and was found to be equal to 9.8374e−007. It is these two regions that correspond to the erroneous regions.

The rest of the regions are classified as correct detection wherein there is a clear demarcation between the two hypotheses. The detection probability is given by the area under the abnormal density function excluding the overlap and was found to be 0.7071. The discriminability is directly proportional to the difference in the means and varies inversely with the variance. Therefore, the farther apart the two density functions are and the lesser the variance they exhibit,

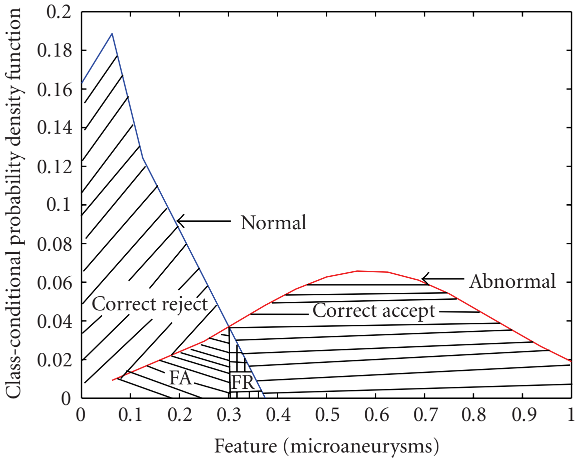

Figure 1: Class-conditional probability density functions for normal category (unaffected retina) and abnormal category (affected retina) with discriminability = 2.7528, false alarm probability = 9.8374e−007, and detection probability = 0.7071.

the greater is the discriminability and the lesser the overlap. If the discriminability is infinity, then the two density functions do not exhibit any overlap and the type-I and type-II errors are zero and the detection is perfect. The discriminability is a quantitative measure of decidability and is independent of the chosen decision criteria. This factor is given as

$$d = \frac{|\mu_1 - \mu_0|}{\sqrt{(\sigma_1^2 - \sigma_0^2)}}. \quad (1)$$

The discriminability for the case shown in Figure 1 was found to be $d = 2.7528$. When a feature value is presented to the system, then it can be classified either as normal or abnormal. This detection process can be treated as a binary-hypothesis testing problem. The hypotheses supported are the null hypothesis, $H_0$ (specifies the absence of the microaneurysms), and the alternative hypothesis, $H_1$ (specifies the presence of the microaneurysms). Each of the hypotheses has a probability density function associated with it. In the case of diabetic retinopathy, each of the two categories, that is, the affected and the unaffected, represents a Gaussian distribution where the feature values tend to a particular value (given by the mean) but with a little variation (given by the variance). For the aforementioned data, maximum likelihood estimation was carried out in order to determine the conditional probability density functions and the problem was reduced to the detection of the following hypotheses:

$$H_0 : p_0 \sim N(\mu_0, \sigma^2) \quad (2)$$

versus

$$H_1 : p_1 \sim N(\mu_1, \sigma^2), \quad (3)$$

where $p_0$ and $p_1$ are the density functions for the normal and the affected case, respectively. The variance obtained for the normal and the abnormal case for the data under consideration was found to be the same. The respective values

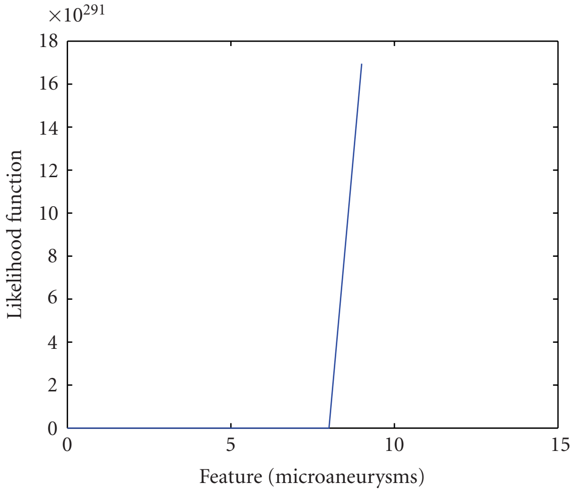

Figure 2: Likelihood ratio $p_1/p_0$ versus feature value (microaneurysms).

obtained for these two sample datasets are $\mu_0 = 0.0475$ and $\mu_1 = 0.5842$ for a standard deviation of $\sigma = 0.0832$. Since the variance for the two categories is the same and the difference between the means is small, the discriminability is small. Decision rule, $\delta$, for $H_0$ versus $H_1$ is a function of the feature and can take either a value of 1 or 0 depending on whether the feature belongs to $H_1$ or $H_0$. Also, if the feature does not belong to $H_1$ then it belongs to $H_0$, that is, $H_0 = H_1^c$. Therefore, the decision rule can be mathematically represented as

$$\delta(x) = \begin{cases} 1 & \text{if } x \in H_1, \\ 0 & \text{if } x \in H_0 = H_1^c, \end{cases} \tag{4}$$

where $x$ is the feature. Detection can be performed by employing any optimal decision criteria. This paper considers the following detection methods.

### 3.1. Likelihood ratio test

The conditional pdf, $p_i(x)$, gives the likelihood that a feature value $x$ belongs to a particular state of nature $H_i$. The likelihood ratio is given by

$$l(x) = \frac{p_1(x)}{p_0(x)}. \tag{5}$$

Detection method that compares the likelihood ratio with a certain threshold value is called the likelihood ratio test. For the Gaussian distributions considered for the two possible classes, the likelihood function is given as

$$l(x) = e^{((\mu_1-\mu_0)/\sigma^2)(x-(\mu_0+\mu_1)/2)}. \tag{6}$$

Figure 2 shows the likelihood ratio. Since the two distributions are independent of each other, the numerator and the denominator can be interchanged.

A threshold $\tau$ is fixed such that decision can be made on $\tau$. Thus, the decision rule becomes

$$\delta(x) = \begin{cases} 1, & l(x) \geq \tau, \\ 0, & l(x) < \tau. \end{cases} \tag{7}$$

The threshold depends on the prior knowledge about each hypothesis and cost or loss associated with each classification. The prior probability does not depend on the feature value. The prior probabilities for diabetic retinopathy can be demographically based on the percentage of population affected by the disease. The sample space consisting of retinal images for our analysis belongs to Louisiana State University Eye Center. The statistics that correspond to Louisiana State University Eye Center imply that 60% of the diabetic population is prone to diabetic retinopathy. Thus, the prior probability for the abnormal case is $P(\text{abnormal}) = 0.6$ and for the normal case would be $P(\text{normal}) = 1 - P(\text{abnormal}) = 0.4$. Loss associated with each classification is determined by a loss matrix $L$, where an element $L_{ij}$ represents the loss associated with choosing a hypothesis $H_i$ when $H_j$ is true. For a binary-hypothesis testing problem, $i$ and $j$ can only take values of 0 or 1. Also, the range of loss would be from 0 to 1. Zero corresponds to no loss and 1 corresponds to maximum loss. Zero loss would be attributed to the case where detection occurs, that is, type-I (FA) and type-II (FR) errors are absent. As mentioned earlier the loss associated with type-II error should be more than the loss associated with type-I error. In other words loss incurred in classifying an abnormal person as normal given by $L_{01}$ should be greater than the converse, that is, $L_{10}$. In our calculations we have made use of the following loss matrix:

$$L = \begin{pmatrix} 0 & 0.8 \\ 0.3 & 0 \end{pmatrix}. \tag{8}$$

The threshold $\tau$ is given as

$$\tau = \frac{P(\text{normal})(L_{10} - L_{00})}{P(\text{abnormal})(L_{01} - L_{11})}. \tag{9}$$

By substituting as follows: $P(\text{normal}) = 0.4$, $P(\text{abnormal}) = 0.6$, $L_{11} = L_{00} = 0$, $L_{01} = 0.8$, and $L_{10} = 0.3$, we get a threshold value of 1.7778. Thus, if the likelihood ratio is more than 1.7778, then the feature should be classified as abnormal, and normal otherwise. Instead of comparing the likelihood ratio with the threshold $\tau$, the feature value can be compared with a threshold $\tau'$ which can be derived from (6). Threshold $\tau'$ was found to be

$$\tau' = \frac{\mu_0 + \mu_1}{2} + \frac{\sigma^2 \log \tau}{\mu_1 - \mu_0}. \tag{10}$$

Thus, the detector becomes

$$\delta(x) = \begin{cases} 1, & x \geq \dfrac{\mu_0 + \mu_1}{2} + \dfrac{\sigma^2 \log \tau}{\mu_1 - \mu_0}, \\ 0, & x < \dfrac{\mu_0 + \mu_1}{2} + \dfrac{\sigma^2 \log \tau}{\mu_1 - \mu_0}. \end{cases} \tag{11}$$

Table 1: Table showing the posterior probabilities for different counts of microaneurysms and the corresponding categorization obtained from the MAP detector.

| Feature value | $P(\text{normal} \mid x)$ | $P(\text{abnormal} \mid x)$ | Classification |
|---|---|---|---|
| 0 | 8.417683e−001 | 1.582317e−001 | Normal |
| 1 | 7.605189e−001 | 2.394811e−001 | Normal |
| 2 | 6.545819e−001 | 3.454181e−001 | Normal |
| 3 | 5.306089e−001 | 4.693911e−001 | Normal |
| 4 | 4.026511e−001 | 5.973489e−001 | Abnormal |
| 5 | 2.866279e−001 | 7.133721e−001 | Abnormal |
| 6 | 1.931653e−001 | 8.068347e−001 | Abnormal |
| 7 | 1.248050e−001 | 8.751950e−001 | Abnormal |
| 8 | 7.826310e−002 | 9.217369e−001 | Abnormal |
| 9 | 4.810631e−002 | 9.518937e−001 | Abnormal |
| 10 | 2.919115e−002 | 9.708088e−001 | Abnormal |
| 11 | 1.756968e−002 | 9.824303e−001 | Abnormal |
| 12 | 1.052091e−002 | 9.894791e−001 | Abnormal |
| 13 | 6.279664e−003 | 9.937203e−001 | Abnormal |
| 14 | 3.740336e−003 | 9.962597e−001 | Abnormal |
| 15 | 2.224729e−003 | 9.977753e−001 | Abnormal |
| 16 | 1.321955e−003 | 9.986780e−001 | Abnormal |

By substituting the appropriate value in the above equation, we get $\tau' = 0.3233$. Thus, if a feature value is above 0.3233, then it can be classified as abnormal, and normal otherwise.

### 3.2. Maximum a posteriori detector

The posterior probability, unlike the prior probability, is the conditional probability that a particular state of nature exists for a given feature value and is represented as $P(H_k \mid x)$, where $k$ represents the class index. For better comprehension, we would represent the posterior probability for the normal category as $P(\text{normal} \mid x)$ and for the abnormal case as $P(\text{abnormal} \mid x)$. In general, the posterior probability is obtained by the Bayes formula as follows:

$$P(H_k \mid x) = \frac{p(x \mid H_k)P(H_k)}{\sum_{l=1}^{c} p(x \mid H_l)P(H_l)}, \tag{12}$$

where $p(x \mid H_k)$ is the class-conditional probability or the likelihood, $P(H_k)$ is the prior probability, and $c$ is the total number of possible hypotheses. The posterior probability for the normal case is thus given as follows:

$$P(\text{normal} \mid x) = \frac{p_0(x) * P(\text{normal})}{p(x)}, \tag{13}$$

where $p_0(x) = p(x \mid H_0)$.

Similarly, the posterior probability for the abnormal case is

$$P(\text{abnormal} \mid x) = \frac{p_1(x) * P(\text{abnormal})}{p(x)}, \tag{14}$$

where $p_1(x) = p(x \mid H_1)$.

Here, $p(x)$ is a scaling factor and is given as

$$p(x) = p_0(x) * P(\text{normal}) + p_1(x) * P(\text{abnormal}). \tag{15}$$

Now, the decision rule is based on choosing that hypothesis whose posterior probability is maximum and hence, it is called maximum a posterior estimator. So, the decision rule is represented as

$$\begin{aligned} &\text{Decide } H_1 \quad \text{if } P(\text{normal} \mid x) > P(\text{abnormal} \mid x), \\ &\text{Decide } H_0 \quad \text{otherwise.} \end{aligned} \tag{16}$$

Classification using MAP detector is depicted in Table 1. It can be deduced from the table that a retinal image that presents four or more microaneurysms is treated as abnormal by the MAP detector. The final diagnosis rests with the physician and is shown in Section 4.

### 3.3. Bayes detector

The Bayes detector is based on the optimality criterion that minimizes the Bayes risk or average risk $r(\delta)$. The Bayes risk is the overall cost incurred by a decision rule $\delta$ and it depends on the conditional risk or expected loss. The conditional risk $R_k(\delta)$ is the average cost incurred by a decision rule when hypothesis $H_k$ is true. Suppose that a particular observed feature is classified as $H_i$, whereas the true hypothesis happens to be $H_j$; then the loss incurred, as shown in the previous section, would be $L_{ij}$. Because $P(H_j \mid x)$ is the probability that the true state of nature is $H_j$, then the expected loss in classifying the feature as $H_i$ would be [23]

$$R_i(\delta) = \sum_{j=1}^{c} L_{ij} P(H_j \mid x), \tag{17}$$

TABLE 2: Table showing the Bayes risk obtained for different counts of microaneurysms and the corresponding categorization.

| Feature value | Classification | Bayes risk | Physician's diagnosis |
|---|---|---|---|
| 0 | Normal | 1.265854e−001 | Normal |
| 1 | Normal | 1.915849e−001 | Normal |
| 2 | Normal | 2.763344e−001 | Normal |
| 3 | Normal | 3.755129e−001 | Normal |
| 4 | Abnormal | 1.207953e−001 | Normal |
| 5 | Abnormal | 8.598838e−002 | Normal |
| 6 | Abnormal | 5.794959e−002 | Abnormal |
| 7 | Abnormal | 3.744149e−002 | Abnormal |
| 8 | Abnormal | 2.347893e−002 | Abnormal |
| 9 | Abnormal | 1.443189e−002 | Abnormal |
| 10 | Abnormal | 8.757346e−003 | Abnormal |
| 11 | Abnormal | 5.270903e−003 | Abnormal |
| 12 | Abnormal | 3.156274e−003 | Abnormal |
| 13 | Abnormal | 1.883899e−003 | Abnormal |
| 14 | Abnormal | 1.122101e−003 | Abnormal |
| 15 | Abnormal | 6.674187e−004 | Abnormal |
| 16 | Abnormal | 3.965865e−004 | Abnormal |

where $c$ is the total number of possible hypotheses. To minimize the overall risk, compute the conditional risk given as above for each possible hypothesis and select that hypothesis for which the risk is minimum. This minimum conditional risk is called the Bayes risk. The average cost incurred by the decision rule when hypothesis $H_0$ is true, that is, the person under consideration is normal, denoted by $R_0(\delta)$, is

$$R_0(\delta) = L_{00}P(\text{normal} \mid x) + L_{10}P(\text{abnormal} \mid x). \quad (18)$$

Similarly, average cost incurred in classifying a person as abnormal is

$$R_1(\delta) = L_{01}P(\text{normal} \mid x) + L_{11}P(\text{abnormal} \mid x). \quad (19)$$

The Bayes risk is given as

$$r(\delta) = \min\left[R_0(\delta), R_1(\delta)\right]. \quad (20)$$

The Bayes decision rule is

$$\begin{aligned} &\text{Decide } H_1 \quad \text{if } R_1(\delta) < R_0(\delta), \\ &\text{Decide } H_0 \quad \text{otherwise.} \end{aligned} \quad (21)$$

The Bayes detector does not provide any condition at the boundary. Therefore, the boundary feature values at which the probability of classifying into each of the categories is the same can be classified into any category. Specifically, at the boundary we should be able to classify each feature as abnormal so that a person for whom the true hypothesis is abnormal is not misdiagnosed.

### 4. EXPERIMENTAL RESULTS

We have made use of 143 retinal images provided by the Louisiana State University Eye Center. Supervised learning was performed for training, whereas unsupervised learning was used to test the system. The system is trained for NPDR. In our experiments we have compared the retinal images of the diabetic patients which do not manifest microaneurysms with those which do. Moderate-to-severe cases were considered for the case wherein microaneurysms are present. A YES decision (abnormal) corresponds to the presence of microaneurysms for the moderate and severe cases of NPDR and a NO decision (normal) relates to the absence of microaneurysms. Each decision has an associated cost that is represented by the Bayes risk. The results obtained are given in Table 2.

Feature value corresponds to the number of microaneurysms exhibited by the test image. The system classifies the affected retinal image as abnormal, whereas unaffected retinal image is classified as normal. The Bayes risk as shown in (20) is given by the minimum of the two expected losses. From Table 1, it can be deduced that for an image that exhibits less than 3 microaneurysms, the expected loss associated with normal classification is less than that with abnormal classification. Hence a feature value of less than 3 has been categorized as normal which is rational. The threshold provided by the likelihood ratio test, that is, $\tau' = 0.3233$, corresponds to a feature value of 4. If the posterior probability of classifying a feature as abnormal is the same as that of normal, then the classification can be made either way. But classifying it as abnormal would reduce the risk involved in misclassification. Thus, if the retinal image presented exhibits 4 or more microaneurysms, it is classified as abnormal. The system classifies a feature value of 4 and 5 as abnormal, whereas the physician's diagnosis is otherwise. This is because of the asymmetric costs associated with each classification. The final decision rests with the physician; therefore, if the system treats a normal image as abnormal the cost incurred is less than that of the converse. The sensitivity of the decision related to the classification of microaneurysms is 100%, while its specificity is 67%. The computational time is mainly dependent on the detection algorithm and it is approximately 10 nanoseconds.

### 5. CONCLUSIONS AND FUTURE WORK

This paper proposed a decision support framework for automated screening of DR for the univariate case. This model can be extended to multiple disorders that would include the covariance associated with all the signs of DR. The experiments support the feasibility of a complete automated screening mechanism that includes all the disorders related to DR. The machine can be made adaptable by including Bayesian learning mechanism that would improve the accuracy of the classifier as a new feature value is presented to it by modifying the priors.

### ACKNOWLEDGEMENTS

Namuduri acknowledges the LSU Health Sciences Division and the NSF Kansas EPSCoR Division for their support in performing the work described in this paper.

### REFERENCES

[1] D. S. Fong, L. Aiello, T. W. Gardner, et al., "Retinopathy in diabetes," *Diabetes Care*, vol. 27, no. suppl 1, pp. S84–S87, 2004.

[2] R. Klein, B. E. Klein, S. E. Moss, M. D. Davis, and D. L. DeMets, "The Wisconsin epidemiologic study of diabetic retinopathy. II. Prevalence and risk of diabetic retinopathy when age at diagnosis is less than 30 years," *Archives of Ophthalmology*, vol. 102, no. 4, pp. 520–526, 1984.

[3] H. Buch, T. Vinding, and N. V. Nielsen, "Prevalence and causes of visual impairment according to World Health Organization and United States criteria in an aged, urban Scandinavian population: the Copenhagen City Eye Study," *Ophthalmology*, vol. 108, no. 12, pp. 2347–2357, 2001.

[4] K. R. Namuduri, H. W. Thompson, and M. E. Hartnet, "A multiscale-multiresolution method for automated retinal feature detection," in *The Association for Research in Vision and Ophthalmology Conference (ARVO '03)*, p. 165, Fort Lauderdale, Fla, USA, May 2003.

[5] The Diabetes Control Complications Trial Research Group, "The effect of intensive treatment of diabetes on the development and progression of long-term complications in insulin-dependent diabetes mellitus," *The New England Journal of Medicine*, vol. 329, no. 14, pp. 977–986, 1993.

[6] UK Prospective Diabetes Study (UKPDS) Group, "Intensive blood-glucose control with sulphonylureas or insulin compared with conventional treatment and risk of complications in patients with type 2 diabetes (UKPDS 33)," *The Lancet*, vol. 352, no. 9131, pp. 837–853, 1998.

[7] MedWeb, "Diabetic retinopathy [Internet]," School of Medicine, University of Birmingham, 2004, http://medweb.bham.ac.uk/easdec/Information_for_patients.html [Accessed 5th June 2005].

[8] S. C. Lee, E. T. Lee, R. M. Kingsley, et al., "Comparison of diagnosis of early retinal lesions of diabetic retinopathy between a computer system and human experts," *Archives of Ophthalmology*, vol. 119, no. 4, pp. 509–515, 2001.

[9] Early Treatment Diabetic Retinopathy Study Research Group (ETDRS), "Grading diabetic retinopathy from stereoscopic color fundus photographs–an extension of the modified Airlie House classification. ETDRS report number 10," *Ophthalmology*, vol. 98, no. 5 suppl, pp. 786–806, 1991.

[10] A. J. Frame, P. E. Undrill, M. J. Cree, et al., "A comparison of computer based classification methods applied to the detection of microaneurysms in ophthalmic fluorescein angiograms," *Computers in Biology and Medicine*, vol. 28, no. 3, pp. 225–238, 1998.

[11] B. M. Ege, O. K. Hejlesen, O. V. Larsen, et al., "Screening for diabetic retinopathy using computer based image analysis and statistical classification," *Computer Methods and Programs in Biomedicine*, vol. 62, no. 3, pp. 165–175, 2000.

[12] L. Gagnon and M.-C. Boucher, "Development of an image analysis software to assist in the diagnostic of diabetic retinopathy," in *Proceedings of the 5th Conference on the Medical Aspect of Telemedicine*, p. 118, Montreal, Quebec, Canada, October 2000.

[13] A. Osareh, M. Mirmehdi, B. T. Thomas, and R. Markham, "Comparative exudate classification using support vector machines and neural networks," in *Proceedings of the 5th International Conference on Medical Image Computing and Computer-Assisted Intervention (MICCAI '02)*, pp. 413–420, Tokyo, Japan, September 2002.

[14] R. Markham, A. Osareh, M. Mirmehdi, B. T. Thomas, and M. Macipe, "Automated identification of diabetic retinal exudates using support vector machines and neural networks," in *The Association for Research in Vision and Ophthalmology Conference (ARVO '03)*, Fort Lauderdale, Fla, USA, May 2003.

[15] C. Serruys, D. Brahmi, A. Giron, et al., "A learning by sample approach for the detection of features in medical images," in *Proceedings of International Conference on Artificial Neural Networks in Medicine and Biology (ANNIMAB-1 '00)*, Göteborg, Sweden, May 2000.

[16] C. I. Sanchez, R. Hornero, M. I. Lopez, and J. Poza, "Retinal image analysis to detect and quantify lesions associated with diabetic retinopathy," in *Ioba*, p. 118, University of Valladolid, Valladolid, Spain, May 2003.

[17] T. Spencer, R. P. Phillips, P. F. Sharp, and J. V. Forrester, "Automated detection and quantification of microaneurysms in fluorescein angiograms," *Graefe's Archive for Clinical and Experimental Ophthalmology*, vol. 230, no. 1, pp. 36–41, 1992.

[18] R. P. Phillips, T. Spencer, P. G. Ross, P. F. Sharp, and J. V. Forrester, "Quantification of diabetic maculopathy by digital imaging of the fundus," *Eye*, vol. 5, part 1, pp. 130–137, 1991.

[19] K. A. Goatman, M. J. Cree, J. A. Olson, J. V. Forrester, and P. F. Sharp, "Automated measurement of microaneurysm turnover," *Investigative Ophthalmology & Visual Science*, vol. 44, no. 12, pp. 5335–5341, 2003.

[20] P. Hänsgen, P. E. Undrill, and M. J. Cree, "The application of wavelets to retinal image compression and its effect on automatic microaneurysm analysis," *Computer Methods and Programs in Biomedicine*, vol. 56, no. 1, pp. 1–10, 1998.

[21] A. M. Mendonça, A. J. Campilho, and J. M. Nunes, "Automatic segmentation of microaneurysms in retinal angiograms of diabetic patients," in *Proceedings of 10th International Conference on Image Analysis and Processing (ICIAP '99)*, pp. 728–733, Venice, Italy, September 1999.

[22] H. V. Poor, *An Introduction to Signal Detection and Estimation*, Springer, New York, NY, USA, 2nd edition, 1994.

[23] R. O. Duda, P. E. Hart, and D. G. Stork, *Pattern Classification*, John Wiley & Sons, New York, NY, USA, 2nd edition, 2000.

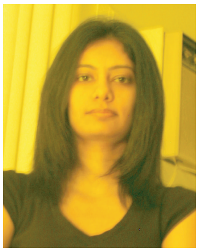

**P. Kahai** received her M.S. degree in electrical and computer engineering from Wichita State University in Spring 2005. Her research interests include network, and statistical pattern recognition. She is currently with the Cisco Systems.

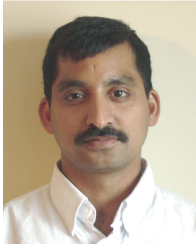

**K. R. Namuduri** received his B.E. degree in electronics and communication engineering from Osmania University, India, in 1984, M. Tech. degree in computer science from University of Hyderabad in 1986, and Ph.D. degree in Computer Science and Engineering from University of South Florida in 1992. He has worked in C-DoT, a telecommunication firm in India, from 1984 to 1986. Currently, he is with the Electrical and Computer Engineering Department at Wichita State University as an Assistant Professor. His areas of research interest include information security, image/video processing and communications, and ad hoc sensor networks.

**H. Thompson** holds a Ph.D. in physiology and a Minor in applied statistics from LSU His primary appointment at the Louisiana State University Health Sciences Center is in the Department of Ophthalmology. He is the Biostatistician for the Ophthalmology Department's NIH Center of Research Excellence Grant, and Director of the LSU Eye Center's Clinical Trials and Biometry Unit. Dr. Thompson is also an Adjunct Associate Professor in the School of Public Health department of Biometry, an Adjunct Associate Professor of Neuroscience, and an Adjunct Associate Professor of Medicine. Dr. Thompson assists many investigators of these departments in various phases of studies including experimental planning, data management, and statistical analysis of the data. His research interests are in the development of statistical methods for the analysis of information in biomedical images and, in particular, in time series of such images.

